# Supplementary material for: Assessment of the viability of photovoltaic system implementation on the New Media Tower of Universitas Multimedia Nusantara using PVSyst software: A feasibility study
Source: PLoS One. 2024 Dec 6;19(12):e0314922. doi: 10.1371/journal.pone.0314922 (PMC11623462; doi:10.1371/journal.pone.0314922)
Supplement: Attachment — Submitted filename: Response to Reviewers.docx [file pone.0314922.s001.docx]

Response to Reviewer PONE-D-24-29663

Assessment of the viability of photovoltaic system implementation on the New Media Tower of Universitas Multimedia Nusantara using PVSyst Software: A feasibility study

Dear Reviewers and Editors,

The authors of this paper greatly appreciate the valuable comments from the reviewers and editors. The suggestions are greatly helpful to improve the quality of our paper. We have made careful modifications strictly according to the Reviewer’s advice. The major modifications to the paper are highlighted in the paper as follows:

Review 1 : Yellow highlight

Review 2 : Green highlight

Response to Reviewer 1

**Comment 1: Reedit the paper based on English language**

Response 1: Thank you for your suggestion. We have revise the paper using Grammarly.

**Comment 2: Vast the introduction section and use some new and related references**

Response 2: We have added the introduction part and also addition references.

Here the added introduction.

In addition, these studies discuss the importance of performance and economic analysis in the implementation of photovoltaic systems to support the transition to renewable energy. Research in Islamabad, Pakistan, using PVSyst to design PV systems in luxury villas and high-rise buildings, revealed that payback analysis and performance ratios are critical to assessing financial viability, both through direct purchase and bank financing [31]. In India, research showed that with government support, residential rooftop PV systems become more economically viable, and simulations using PVSyst compared different PV module technologies based on energy performance and long-term economic analysis [32]. Research in Bangladesh emphasized the efficiency and carbon emission reduction potential with grid-connected PV systems, where the systems have the potential for cost savings and generate revenue from selling electricity to the grid [33]. The findings suggest that a comprehensive technical and economic feasibility analysis prior to PV installation is essential to ensure sustainable energy benefits in various locations. Based on this research, we propose a research related to the feasibility study of the photovoltaic system at the New Media Tower at Universitas Multimedia Nusantara using PVSyst. This analysis will include technical and economic evaluations.

Although Universitas Multimedia Nusantara (UMN) has implemented the green building concept in Building C through the use of double facades that help reduce energy consumption, efforts to achieve more holistic sustainability can still be improved by utilizing renewable energy, especially solar energy. Currently, there is no comprehensive study on the potential of solar energy on UMN's campus, especially regarding the installation of photovoltaic systems in Building C. To support the target of reducing the carbon footprint and increasing energy efficiency in this building, research is needed on the technical and financial feasibility of a solar power generation system. This research is also important to determine the most optimal location around Building C that can produce maximum energy, and evaluate whether the target of providing 20% of the building's electricity needs through solar power can be achieved.

The main focus of this research is on the technical and financial feasibility study of a solar power generation system in Building C or New Media Tower, Universitas Multimedia Nusantara (UMN) using PVSyst. The objective is to evaluate the potential locations around the building for solar panel installation. The target of this research is to fulfill 20% of Building C's electricity demand through solar energy. Several locations around Building C will be analyzed to assess the technical and financial feasibility of installing a solar power generation system, making it possible to achieve the target of providing 20% of the building's electricity needs from solar power. Furthermore, this research will provide an in-depth analysis of the technical and financial feasibility of installing photovoltaic systems at selected locations in and around UMN Building C.

The novelty of this research lies in the integration of renewable energy technology, especially photovoltaic systems or solar power plants, in the concept of green building in tropical areas, with objects in university buildings in Indonesia, especially in UMN. The use of PVSyst simulation software to evaluate the solar energy potential of UMN's Building C is an approach that has not been widely applied in Indonesian educational institutions. This research also offers results that allow implementation to achieve energy reduction targets through technical and economic approaches.

**Comment 3: Compare the results with other similar researches**

Response 3: In this study,

- The rooftop PV system in UMN produced about 202 MWh/year, close to the target of 209.64 MWh/year, while other studies in locations with higher solar radiation reported production above 250 MWh/year.
- The efficiency of the system in the parking lot is only 3.38 kWh/kWp/day, 15% lower than the rooftop (3.98 kWh/kWp/day). Other studies have shown a 10-20% drop in efficiency in shaded locations.
- The study showed a payback period of 7.1 years and an ROI of 115.8% in 20 years, which is in line with other studies reporting payback periods between 5 to 10 years.

**Comment 4: What is the primary objective of this study?**

Response 4: The objective is to evaluate the potential locations around the building for solar panel installation. The target of this research is to fulfill 20% of Building C's electricity demand through solar energy. Several locations around Building C will be analyzed to assess the technical and financial feasibility of installing a solar power generation system, making it possible to achieve the target of providing 20% of the building's electricity needs from solar power. Furthermore, this research will provide an in-depth analysis of the technical and financial feasibility of installing photovoltaic systems at selected locations in and around UMN Building C.

**Comment 5: Why is addressing climate change urgent according to the paper?**

Response 5: Mitigating climate change is critical because it causes emissions of greenhouse gases such as carbon dioxide (CO_2_). Emissions will accelerate global warming and negatively impact the environment. If not addressed, climate change will cause greater disruption to ecosystems, economies and human life, including risks to global health and safety. Therefore, the implementation of sustainable solutions, such as the use of renewable energy through photovoltaic systems, is necessary to reduce the carbon footprint and mitigate the impacts of climate change.

**Comment 6: What are green building practices, and how do they contribute to sustainability?**

Response 6: Green building practices are an approach to building design, construction and maintenance that focuses on reducing environmental impact and conserving resources. These practices include energy efficiency. Some examples are the use of energy-efficient technologies such as solar panels, water conservation with rainwater harvesting systems, and the use of sustainable materials that can be renewed or recycled. In addition, green buildings also improve indoor air quality which affects the occupants. By reducing energy consumption, waste and emissions, green building practices contribute to long-term sustainability.

**Comment 7: What are the main features of Building C that align with green building principles?**

Response 7: The main features of the C UMN building that are in line with green building principles are the availability of many windows for natural lighting and double facades so that the lighting entering the room is not excessive. On the 3^rd^ floor of Building C, there is also a garden planted with greenery. In addition, there is also a hole which is a light well where the light well will deliver light to the 1st floor of building C so that it will minimize the use of artificial light.

Figure of double façade or secondary skin in C UMN building

Figure of window and double façade or secondary skin in C UMN building from classroom

C UMN building

Figure of light well in the 3^rd^ floor of Building C

D UMN building

C UMN building

C UMN building

Figure of C UMN building from 3rd floor and 1st floor

**Comment 8: How does the double facade of Building C contribute to energy efficiency?**

Response 8: Building C's double façade improves energy efficiency by regulating the amount of sunlight entering the building, thereby reducing the need for artificial lighting and cooling. The perforated double façade helps retain natural lighting while minimizing heat gain, ensuring thermal comfort for occupants. This design reduces the building's reliance on active cooling systems and artificial lighting, resulting in significant energy savings.

**Comment 9: What role does solar energy play in reducing carbon footprints?**

Response 9: Solar energy plays a role in reducing the carbon footprint by generating electricity without producing greenhouse gas emissions. Unlike fossil fuels, solar power utilizes energy from the sun, which is a renewable resource. It can fulfill energy needs with minimal contribution to air pollution or carbon dioxide release.

**Comment 10: Why are simulations using software like PVSyst essential before actual PV system installation?**

Response 10: Th Simulation using software such as PVSyst is important before installing a photovoltaic system as it allows accurate prediction of the system's performance, efficiency, and energy output. These simulations help assess various factors such as solar irradiation, optimal panel placement, so that it will be more well-planned. In addition, PVSyst also provides financial analysis, helping to evaluate the economic viability of the system and estimate the payback period, ensuring the right decisions are made before the actual investment.

**Comment 11: What are the potential locations for PV installation on Building C?**

Response 11: Potential locations for photovoltaic installation in Building C include the roof, outer walls, and areas around the building. On the roof of the building there is ample space for solar panels with maximum sun exposure. The outer walls of the building, especially those exposed to direct sunlight, can also be utilized for vertical installations. In addition, areas around Building C, such as open spaces or parking lots, can be evaluated for ground-mounted solar panels.

**Comment 12: Why is the rooftop of Building C considered the most suitable site for PV installation?**

Response 12: On the roof of the building there is ample space for solar panels with maximum sun exposure.

**Comment 13: What are the challenges associated with installing PV systems on the rooftop of Building C?**

Response 13: Installing a photovoltaic system on the roof of Building C presented several challenges. First, the structural integrity of the roof had to be evaluated to ensure that it could support the additional load of the solar panels and their mounting systems without compromising the safety of the building. In addition, safe access for maintenance and cleaning needed to be considered due to the location of the rooftop-mounted photovoltaic system.

**Comment 14: How does shading affect the performance of PV systems?**

Response 14: Shadows on solar panels will reduce the performance of the photovoltaic system, resulting in reduced electrical energy gain. Even partial shading can also decrease the energy output, as PV cells are connected in series, meaning that the performance of one shaded cell can affect the entire system. This reduces the overall efficiency, power production, and financial returns of the photovoltaic system.

**Comment 15: What are the advantages and disadvantages of installing PV systems in the outdoor motorcycle parking area?**

Response 15: Advantages: the photovoltaic system can also act as a roof for parked motorcycles, disadvantages: there are several trees and buildings nearby that can cause shading effects.

**Comment 16: What types of solar panels and inverters were used in the simulations?**

Response 16: In this study, Canadian Solar CS3K-290P 290 Watt poly Solar Panel Module was used. This panel is known as a polycrystalline panel with a capacity of 290 Watts which is used to maximize the absorption of solar energy. Meanwhile, the inverter used is a Ginlong technologies 30kW 200-800V TL 50/60 Hz Solis-30K inverter, which has an output voltage of 200-800V. This inverter functions to convert direct current (DC) generated by solar panels into alternating current (AC) which can be used by the building's electrical network.

**Comment 17: How was the optimal orientation for PV panels determined in the study?**

Response 17: For the simulation on the roof of the building, two types of orientation were used, Orientation 1: tilt 15°, azimuth 10° and Orientation 2: tilt 30°, azimuth 10°. For the simulation in the parking lot area, where there is already a suitable roof for installation, only one orientation was used, with a tilt of 15° and an azimuth of 70°.

**Comment 18: What are the key findings from the performance simulations of the rooftop and parking area PV systems?**

Response 18:

Rooftop System: Simulation results show that rooftop systems have better performance with fewer losses, especially related to shadows. The optimized orientation of solar panels on the roof helps maximize energy absorption, minimize shadow effects, and improve system efficiency.

Parking Lot Systems: While the parking lot system remained productive, it experienced greater shadow losses due to the lower placement of the panels and their proximity to surrounding objects, as seen in the nearby shadow scheme. This leads to a decrease in energy output compared to the rooftop system.

**Comment 19: How does the financial analysis demonstrate the viability of the rooftop PV system?**

Response 19: The financial analysis shows that the PV system project on the roof of Building C is financially viable with several important indicators. First, the payback period is reached within 7.1 years, which means that the entire investment cost will be recovered within that period. Second, after 20 years of operation, the project is estimated to generate a profit of Rp1.4 billion, with a return on investment (ROI) of 115.8%. This shows that the project is not only able to recover the investment costs in a relatively short period of time, but also generate significant profits in the long run, making it worth implementing.

**Comment 20: What is the payback period for the rooftop PV system, and why is it significant?**

Response 20: the payback period is reached within 7.1 years, which means that the entire investment cost will be recovered within that period. This shows that the project is not only able to recover the investment costs in a relatively short period of time, but also generate significant profits in the long run, making it worth implementing.

**Comment 21: How does the return on investment (ROI) for the rooftop PV system compare to the initial investment?**

Response 21: after 20 years of operation, the project is estimated to generate a profit of Rp1.4 billion, with a return on investment (ROI) of 115.8%. This shows that the project is not only able to recover the investment costs in a relatively short period of time, but also generate significant profits in the long run, making it worth implementing.

**Comment 22: What assumptions were made in the economic analysis of the PV system project?**

Response 22: Economic analysis of PV system projects:

The project is assumed to have a useful life of 20 years, starting in 2024.

The inflation rate was assumed to be 3%, and the discount rate was set at 0%, indicating no adjustment for the time value of money.

It is assumed that the investment is fully funded by Universitas Multimedia Nusantara (UMN) itself, so no loan interest is calculated.

Savings on electricity bills are calculated using the feed-in tariff, with off-peak (LWBP) electricity priced at Rp1,035.78/kWh and peak load (WBP) electricity priced at Rp1,553.67/kWh. However, as the system does not include energy storage, all electricity production is priced at the off-peak rate, as production only occurs during the day.

Annual operating costs include maintenance, salaries, service charges, cleaning, and administration, totaling IDR 66,000,000 per year.

**Comment 23: How does the study address the administrative challenges of installing PV systems on the rooftop?**

Response 23: This research addresses the administrative challenges of installing PV systems by incorporating licensing and administrative costs into the economic analysis. In addition, the importance of planning and coordination with relevant parties to ensure regulatory compliance was highlighted. This helps to reduce administrative barriers during the installation process.

**Comment 24: What are the environmental benefits of implementing the rooftop PV system at UMN?**

Response 24: Reducing dependence on fossil energy and lowering greenhouse gas emissions, improving energy use efficiency with local electricity production, and supporting sustainability by utilizing renewable energy sources.

**Comment 25: How does the study contribute to sustainable energy initiatives in academic institutions?**

Response 25: This study supports sustainable energy initiatives in academic institutions by analyzing the implementation of solar panel systems on the roof of Universitas Multimedia Nusantara (UMN). This research demonstrates the potential carbon emission reduction and energy efficiency that can be achieved through the use of renewable energy. In addition, the results can serve as an example for other institutions to integrate green technologies.

**Comment 26: What are the limitations of the study, and how might they impact the results?**

Response 26: Limitations of this study include the specific location for the photovoltaic (PV) system assessment, which is limited to the New Media Tower at Universitas Multimedia Nusantara, and the reliance on solar irradiation data from the Meteonorm database from 2016 to 2021. In addition, the simulation was conducted using PVSyst software, which may not account for all variables in the real world. These limitations may impact the accuracy of the performance and economic analysis, as local climate variations and site-specific conditions may not be fully captured, potentially leading to overestimated or underestimated energy generation and financial feasibility.

**Comment 27: What future research directions does the paper suggest?**

Response 27:

- Can incorporate energy storage solutions to improve PV system reliability and efficiency, especially during peak load hours.
- Conduct studies to monitor the actual performance of installed PV systems over time, assessing their effectiveness and reliability under different environmental conditions.
- Expanding the study to include other buildings and locations within the campus of Universitas Multimedia Nusantara or other institutions to compare performance metrics and economic feasibility in different places.

Response to Reviewer 2

**Comment 1: It is suggested that the text be revised by a native in English language.**

Response 1: Thank you for your suggestion. We have revise the paper using Grammarly.

**Comment 2: Abstract. Please provide more details (numerical results) in the abstract section.**

Response 2: We have added the numerical results in the abstract.

**Before**

The urgency of addressing climate change, exacerbated by greenhouse gas emissions, necessitates sustainable solutions, including green building practices and renewable energy adoption. This study focuses on the feasibility of implementing solar photovoltaic (PV) systems at Universitas Multimedia Nusantara (UMN), particularly on Building C, known as the New Media Tower, designed with green building principles. Solar energy, an increasingly prominent renewable source, presents a viable solution to reduce carbon footprints. Before actual installation, thorough simulations using software like PVSyst are essential to predict energy output and evaluate system efficiency. Several studies have explored PV system feasibility using simulations, highlighting the importance of software selection for accurate assessments. Building C offers potential locations for PV installation, with the rooftop being a primary consideration due to its expansive area and minimal shading. Despite administrative challenges and the unconventional rooftop design, it emerges as the most suitable site for PV installation to meet electricity demands. Conversely, outdoor motorcycle parking areas present affordability and accessibility advantages but are constrained by shading from surrounding structures and limited space. Technical evaluations reveal that only the rooftop PV system meets electricity generation targets, highlighting its superiority over the parking area system. Financial analysis demonstrates the rooftop system's viability, with a payback period of 8.2 years and a significant return on investment (ROI). Although the upfront investment is substantial, the long-term benefits justify implementation. Overall, this study underscores the technical and financial feasibility of rooftop PV systems on Building C at UMN, offering valuable insights for sustainable energy initiatives in academic institutions.

**After**

The urgency of addressing climate change, exacerbated by greenhouse gas emissions, necessitates sustainable solutions, including green building practices and renewable energy adoption. This study focuses on the feasibility of implementing solar photovoltaic systems at Universitas Multimedia Nusantara (UMN), particularly on Building C, known as the New Media Tower, designed with green building principles. Solar energy, an increasingly prominent renewable source, presents a viable solution to reduce carbon footprints. Before actual installation, thorough simulations using software like PVSyst are essential to predict energy output and evaluate system efficiency. Several studies have explored PV system feasibility using simulations, highlighting the importance of software selection for accurate assessments. Building C offers potential locations for PV installation, with the rooftop being a primary consideration due to its expansive area and minimal shading. The rooftop PV system simulation shows an annual energy production of 202 MWh, close to the target of 209.64 MWh, while the parking area system only generates 64.5 MWh/year. Technical evaluations reveal that only the rooftop PV system meets electricity generation targets, highlighting its superiority over the parking area system. Financial analysis demonstrates the rooftop system's viability, with a payback period of 8.2 years and a return on investment (ROI) of 115.8%. Although the upfront investment is substantial, the long-term benefits justify implementation. Overall, this study underscores the technical and financial feasibility of rooftop photovoltaic systems on Building C at UMN, offering valuable insights for sustainable energy initiatives in academic institutions.

**Comment 3: Keywords. Avoid abbreviations in this section.**

Response 3: Thank you. We have made a revision on the abstract part.

Before revision, **Keywords:** Climate Change, Green Building, Carbon Footprint, PVSyst, ROI

After revision, **Keywords:** Climate Change, Green Building, Carbon Footprint, PVSyst software, return on investment

**Comment 4: Introduction. I believe that the introduction needs to be changed. Introduction should be rewritten focusing on the aim of your work. The statement of the issue and research background is not well done.**

Response 4: Thank you for the comments. Here the revised paragraph.

**Before**

Several locations around Building C UMN will be evaluated for potential installation of PV systems. This activity aims to assess the feasibility of these locations, assuming a target of supplying 20% of the electricity used by Building C UMN with solar power. The objective of this research is to evaluate the locations for installing photovoltaic systems on Building C UMN. Subsequently, the technical and financial feasibility of installing photovoltaic systems at locations on and around Building C on the Universitas Multimedia Nusantara campus will be analyzed.

**After revised**

Although Multimedia Nusantara University (UMN) has implemented the green building concept in Building C through the use of double facades that help reduce energy consumption, efforts to achieve more holistic sustainability can still be improved by utilizing renewable energy, especially solar energy. Currently, there is no comprehensive study on the potential of solar energy on UMN's campus, especially regarding the installation of photovoltaic systems in Building C. To support the target of reducing the carbon footprint and increasing energy efficiency in this building, research is needed on the technical and financial feasibility of a solar power generation system. This research is also important to determine the most optimal location around Building C that can produce maximum energy, and evaluate whether the target of providing 20% of the building's electricity needs through solar power can be achieved.

The main focus of this research is on the technical and financial feasibility study of a solar power generation system in Building C or New Media Tower, Universitas Multimedia Nusantara (UMN). The objective is to evaluate the potential locations around the building for solar panel installation. The target of this research is to fulfill 20% of Building C's electricity demand through solar energy. Several locations around Building C will be analyzed to assess the technical and financial feasibility of installing a solar power generation system, making it possible to achieve the target of providing 20% of the building's electricity needs from solar power. Furthermore, this research will provide an in-depth analysis of the technical and financial feasibility of installing photovoltaic systems at selected locations in and around UMN Building C.

**Comment 5: Introduction. More relevant articles with their most important results should be mentioned.**

Response 5: We have added the three articles in the paper, but we also added again the threes new relevant articles such as is attached here.

Added paragraph that explain the relevant articles.

In addition, these studies discuss the importance of performance and economic analysis in the implementation of photovoltaic systems to support the transition to renewable energy. Research in Islamabad, Pakistan, using PVSyst to design PV systems in luxury villas and high-rise buildings, revealed that payback analysis and performance ratios are critical to assessing financial viability, both through direct purchase and bank financing [31]. In India, research showed that with government support, residential rooftop PV systems become more economically viable, and simulations using PVSyst compared different PV module technologies based on energy performance and long-term economic analysis [32]. Research in Bangladesh emphasized the efficiency and carbon emission reduction potential with grid-connected PV systems, where the systems have the potential for cost savings and generate revenue from selling electricity to the grid [33]. The findings suggest that a comprehensive technical and economic feasibility analysis prior to PV installation is essential to ensure sustainable energy benefits in various locations. Based on this research, we propose a research related to the feasibility study of the photovoltaic system at the New Media Tower at Universitas Multimedia Nusantara using PVSyst. This analysis will include technical and economic evaluations.

**Comment 6: Introduction. The novelty of the work must be written on the more clear and more concise way at the end of introduction section.**

Response 6: The novelty has been added based on the comments. Here the additional paragraph related to the novelty explanation.

**Paragraph added**

The novelty of this research lies in the integration of renewable energy technology, especially photovoltaic systems or solar power plants, in the concept of green building in tropical areas, with objects in university buildings in Indonesia, especially in UMN. The use of PVSyst simulation software to evaluate the solar energy potential of UMN's Building C is an approach that has not been widely applied in Indonesian educational institutions. This research also offers results that allow implementation to achieve energy reduction targets through technical and economic approaches.

**Comment 7: Methods. Methods is incompletely written and needs to be completed according to the relevant sources.**

Response 7: This is the additional description of methods.

**Methods**

This section describes the methodology used for the technical and economic analysis of the solar photovoltaic (PV) system on Building C at Universitas Multimedia Nusantara (UMN). The analysis process was carried out in two major phases: technical simulations using PVSyst software and economic evaluation to assess project feasibility.

**Data Collection**

Initially, data collection involved a comprehensive site survey of Building C and its surroundings. Key data included the roof area, solar irradiance, and shading analysis. The survey also covered information on existing infrastructure that may impact PV system installation, such as HVAC systems, antennas, and other equipment already present on the roof. This information was critical for system orientation and shading simulations.

For solar irradiance data, historical meteorological data for the region was obtained through the Meteonorm database, which provides location-specific irradiance values necessary for accurate simulations. The survey also took into account local weather conditions, including temperature and wind speeds, which affect PV system performance.

**System Design and Simulation Using PVSyst**

Once the site-specific data was gathered, system design and simulations were carried out using PVSyst software, a widely recognized tool for PV system design and energy yield prediction. The following steps were taken:

- Defining PV System Parameters
- Array Sizing
- Shading Analysis
- System Orientation
- Energy Yield Calculation

**Economic Analysis**

To evaluate the financial feasibility of the rooftop PV system, an economic analysis was conducted based on the following steps:

- Cost Estimation
- Operational Costs
- Revenue Assumptions
- Net Present Value (NPV) Calculation
- Payback Period and ROI

**Comment 8: Methods. Calculations should be mentioned in detail. Only the software is mentioned and there is no method.**

Response 8: The method of calculation analysis is included in Economic Analysis part, that is

Cost Estimation, Operational Costs, Revenue Assumptions, Net Present Value (NPV) Calculation, Payback Period and ROI

**Comment 9: Methods. Details of economics analysis should be mentioned.**

Response 9: Thank you for the suggestion. We have mentioned the economic analysis in the article.

**Comment 9: Result and discussion. The results is generally poorly explored. This section should be redesigned in order to explore and discuss the main findings with other international similar works. Also the discussion should be improved.**

Response 9: Thank you for the suggestion. We have added the discussion related to the main finding in this work

Added paragraph

Based on the result and discussion in this study, the rooftop PV system in UMN produced about 202 MWh/year, close to the target of 209.64 MWh/year, while other studies in locations with higher solar radiation reported production above 250 MWh/year. The efficiency of the system in the parking lot is only 3.38 kWh/kWp/day, 15% lower than the rooftop (3.98 kWh/kWp/day). Other studies have shown a 10-20% drop in efficiency in shaded locations. The study showed a payback period of 7.1 years and an ROI of 115.8% in 20 years, which is in line with other studies reporting payback periods between 5 to 10 years.
